# A Tailored and Engaging mHealth Gamified Framework for Nutritional Behaviour Change

**DOI:** 10.3390/nu15081950

**Published:** 2023-04-18

**Authors:** Silvia Orte, Carolina Migliorelli, Laura Sistach-Bosch, Meritxell Gómez-Martínez, Noemi Boqué

**Affiliations:** 1 Unitat de Salut Digital, Centre Tecnològic de Catalunya, Eurecat, 08005 Barcelona, Spain; 2 Unitat de Nutrició i Salut, Centre Tecnològic de Catalunya, Eurecat, 43204 Reus, Spain

**Keywords:** personalized nutrition, behaviour change, gamification, health promotion, mobile health

## Abstract

Mobile health applications (apps) have been shown to be effective for improving eating habits. However, most of the existing apps rely on calorie and nutrient counting which have several limitations including the difficulty in sustaining long-term use, inaccuracy, and the risk of developing eating disorders. We designed and developed a mHealth framework for nutritional behaviour change, integrated into the CarpeDiem app, that focuses on the intake of key food groups which are known to have a higher impact on health indicators instead of the intake of nutrients. This framework is mainly based on a gamified system that delivers personalized dietary missions to the user and provides motivational recommendations that help the user to achieve these missions. Its design was guided by an evidenced-based theory of behavioural change, the HAPA model, and it is also characterized by the personalization of the system and the use of a recommender system based on advanced artificial intelligence techniques. Overall, the approach used in the present app could foster a sustained improvement of eating habits among the general population, which is the main challenge of dietary interventions, decreasing the risk of developing the chronic diseases associated with unhealthy dietary habits.

## 1. Background

Noncommunicable diseases (NCDs) are considered to be a major public health challenge [1]. The prevention and control of NCDs involve the promotion of healthy behaviours since the majority of these diseases are driven by modifiable risk factors including diet and physical activity [2]. However, interventions aimed at health promotion are challenging mainly because humans are hardwired to resist change. In this sense, behavioural change techniques (BCTs) have been identified as a crucial strategy to facilitate the acquisition of new healthy habits [3]. Meanwhile, mobile health (mHealth) has emerged as an effective tool for implementing BCTs, owing to its interactive and adaptable nature, as well as the widespread availability of mobile phones. With its ability to engage users and deliver personalized interventions, mHealth represents an effective technology for health promotion.

BCTs are particular tactics that can be employed to encourage a shift in healthy habits. They are often used in the development of behaviour change interventions, and they aim to target different aspects of behaviour, such as motivation, self-regulation, and environmental influences. Some BCTs include goal setting, self-monitoring, social comparison, social support, and rewards [4]. These are often grouped into frameworks that provide a systematic approach to behaviour change. Some of the most commonly used frameworks for promoting healthy habits include the Transtheoretical Model (TTM) [5], Social Cognitive Theory (SCT) [6], Health Belief Model (HBM) [7], and Health Action Process Approach (HAPA) [8]. While all of these models share the goal of promoting behaviour change, they differ in their approach and focus. For example, the TTM emphasizes the cyclical nature of behaviour change and the need for different strategies at different stages of the process. SCT focuses on the importance of personal, environmental, and behavioural factors in promoting behaviour change. The HBM emphasizes the role of beliefs and perceptions in shaping behaviour, and the HAPA model provides a comprehensive approach to behaviour change by addressing both the motivational and volitional aspects of the process. Each of these strategies offers a unique perspective on behaviour change, and the choice of each one depends on the specific behaviour and population being targeted.

Smartphones have become increasingly popular for delivering behaviour change interventions due to their widespread availability and accessibility. Many BCTs can be effectively delivered through mHealth platforms, either as stand-alone interventions or as part of a larger program. These platforms, compared to in-person interventions, increase the personalisation of the intervention as well as the possibility of obtaining real-time feedback, social comparison, and self-monitoring [9,10]. Moreover, there is no need for real-time interaction between healthcare professionals and the final users. Different meta-analyses and systematic reviews [11,12] have revealed that mHealth applications (apps) may be effective for improving eating habits and diet quality and for disease prevention although their long-term impact remains to be elucidated.

Most available mHealth apps for the general population are focused on weight management and use mainly self-monitoring of food intake and physical activity. Some of them also include health recommender systems and have considerable potential for behaviour change and achieving a healthier lifestyle [13]. Personalized recommendations have a significantly stronger impact on users’ behaviour than do generic ones [14]. Recommender systems uses algorithms to help users adopt better habits by recognizing specific profile indications and tailoring the interventions accordingly. Thus, recommendation systems are considered an effective technology to aid users in adjusting their eating behaviour and could help users to make better choices for achieving a healthier diet and lifestyle.

In this paper, we present a mHealth framework for nutritional behaviour change that has been developed as part of the CarpeDiem app, whose general characteristics have been previously published [15]. Briefly, the CarpeDiem app is aimed at promoting healthy habits among the general population by working on the main pillars of health: nutrition, physical activity, and sleep. It consists of an advanced interface that assists and entertains users, providing them with education on healthy habits, triggering alarms and nudges, and motivating them to comply with personalized goals. CarpeDiem aims at adapting, personalising, and delivering motivational, informative, and gamified strategies through an artificial intelligence (AI)-based recommender system called BECOME (behaviour Change recOMender systEm) [16]. The artificial intelligence is based on a holistic model that conforms to a virtual representation of the users and is created through rigorous profiling algorithms, which considers the main domains associated with healthy habits. Here, we focus on the design and development of the nutrition domain, which by using a gamified approach, delivers personalized dietary missions focused on key food groups to the users. This nutritional behaviour change framework has been built through the implementation of the HAPA model for fostering behaviour change, the personalization of the system, and the use of a recommender system based on advanced AI techniques.

### Related Work

In recent years, various nutrition tracking techniques have grown in popularity because they help individuals to self-monitor and improve their dietary habits. Over the past years, several innovative approaches have been published, each with its own advantages and disadvantages. The promotion of healthy eating habits is crucial for improving diet-related health risk factors and preventing chronic diseases although a considerable gap exists in the research since most of the interventions have focused on specific audiences (e.g., overweight or obese adults [17,18], individuals with diabetes [19,20], or cancer patients [21]) or examine single nutrition-related health outcomes (e.g., weight loss [22], diabetes [23,24], or glycaemic control [25]).

mHealth apps have proven to be effective in inducing positive dietary changes [11]. However, most of these apps are mainly based on diet monitoring by tracking calories and nutrients. Although food tracking has been shown to improve adherence to healthy dietary habits and promote weight loss, there are several disadvantages associated with its use. First, food tracking requires the manual entry of food intake daily, and this is time- and effort-consuming and unsustainable in the long-term. Second, the accuracy of the nutritional intake data obtained through these apps is usually low due to the limited databases, and the multicultural properties of foods and issues of self-reporting, among other issues, are not considered [26]. Third, the use of calorie-counting and body-weight tracking has raised alarming concerns due to its relation to the development of eating disorders such as anorexia or binge-eating disorder [27,28].

While in recent years, new methods based on automated food recognition have been employed for mobile diet tracking, this is still an in-progress technology whose validity is far from the ideal [29]. One notable technique is the use of image recognition to identify and estimate portion sizes of food items, as listed in the review paper of Tahir and Loo, 2021 [30]. These methods have the advantage of being user-friendly and efficient, as individuals can simply take a picture of their meal to receive nutritional information. However, limitations include potential inaccuracies in portion estimation and difficulties in recognizing mixed or processed foods. Furthermore, to have sufficient accuracy, multiview-image methods are necessary. These methods require complex processing, images from different angles, and calibration objects, which reduce user retention and increase the tedium for elderly users.

Another approach is the implementation of wearable devices to track the consumption of nutrients and calories [31,32]. These devices provide continuous, real-time feedback to users, promoting greater awareness of their nutritional intake. However, they may suffer from inaccuracies due to individual differences in metabolism and potential user discomfort from wearing such devices. Furthermore, as these systems are in their nascent stages and not yet commercially available, their widespread availability and long-term effectiveness remain to be seen.

For these reasons, to design the CarpeDiem app we relied upon a different approach by focusing on a more qualitative dietary assessment based on a simplified FFQ, from which we can deliver personalised dietary missions to the user with the objective of working at the food groups level instead of at the nutrient level. This is a way of bringing the recommendations from dietary guidelines closer to the population in a more personalized, interactive, and engaging manner. Since this approach avoids the continuous need to fill in the data through the smartphone and therefore is less invasive, while it is more flexible in avoiding nutrient- and calorie-counting, we think it can bring better outcomes and adherence, especially in the long-term. This can minimise the risk of generating any type of obsessive behaviour, and is thus suitable for users at risk of developing eating disorders, as well as older and less tech-savvy individuals who have no interest in constantly monitoring themselves with a tool. This simplicity also provides the advantage of being fully adaptable to working with more advanced monitoring techniques such as the aforementioned automated food recognition and wearable nutrition devices. For instance, future missions could include taking photos of food items or achieving some nutrition goals measured through a wearable, which would then improve recommendations through a reinforcement learning system.

## 2. Materials and Methods

The objective of the mHealth framework for nutritional behaviour change, developed within the CarpeDiem app, is to promote the adoption of a balanced and healthy diet by fostering a dietary behaviour change that triggers the acquisition of key healthy dietary habits among the general population in order to prevent the development of chronic diseases. Specifically, it has the following features:

- It considers dietary guidelines regarding the consumption of key food groups.
- It contains a gamified system based on custom dietary missions.
- It has a pool of motivational recommendations for each mission.
- It is enhanced with a recommender system based on advanced AI techniques.
- It is based on the HAPA Model, a psychosocial theory of health behaviour change that describes how to persuade people to adopt new behaviours.
- It has a user interface properly designed to minimise the actions to be carried out by the user for the introduction of information.
- It is designed and implemented by an interdisciplinary team including nutritionists, data analysts, designers, and developers.

The design was based on the dietary guidelines “Small changes to eat better” from the Public Health Agency of Catalonia [33], which is an educational tool that helps to identify key dietary aspects that, with small modifications, can significantly improve the quality and sustainability of the diet. Briefly, the main dietary changes proposed in this guide are to eat more fruit and vegetables, pulses, and nuts; to switch to water, wholegrains, virgin olive oil, and seasonal and locally grown foods; and to eat less salt, sugar, red and processed meats, and ultraprocessed food.

### 2.1. Behaviour Change Methodology

The HAPA model has gained recognition as an effective theoretical framework for understanding and designing interventions that promote health behaviour change. HAPA distinguishes between two phases of behaviour change: the motivational phase and the volitional phase. The motivational phase includes preintentional processes such as risk perception, outcome expectancies, and self-efficacy, while the volitional phase focuses on the processes that facilitate or impede the translation of intentions into action [34]. The HAPA model highlights the importance of self-regulatory processes such as planning, goal-setting, and coping strategies in the volitional phase of behaviour change. Compared to other models, HAPA provides a more comprehensive and systematic approach to understanding the determinants of health behaviour change. It offers a structured and integrated framework that captures both the motivational and volitional phases of behaviour change. Additionally, HAPA recognizes the role of contextual and individual factors in influencing health behaviours, such as personality traits and environmental factors. This is particularly important for designing interventions for health behaviour change as it allows for the development of tailored and personalized interventions that address the unique needs of individuals [8]. The application of HAPA in the design of the current gamified system to track and improve nutritional behaviours offers several advantages to support the translation of intentions into action. HAPA provides a theoretical foundation for the integration of game elements both in the motivational and volitional phases of behaviour change. During the motivational phase, the user receives feedback on their nutritional status, which enhances the user’s risk perception. Personalized missions are then gradually adjusted to help the user achieve an improvement in that behaviour, increasing self-efficacy. Additionally, by receiving personalized recommendations, the user can understand the potential outcomes of both unhealthy behaviours and the positive changes that could result from modifying habits. In the volitional phase, the user has the freedom to select which mission to focus on each week, enabling goal-setting and planning. The missions not only aim to change specific behaviours (e.g., start eating more unsalted natural nuts) but also to guide users in planning the necessary actions required to achieve these goals (e.g., go to the grocery store to buy unsalted natural nuts). Coping strategies are also incorporated into the system to support users if they encounter difficulties with their missions, allowing them to change to more motivating tasks.

**Figure 1:**
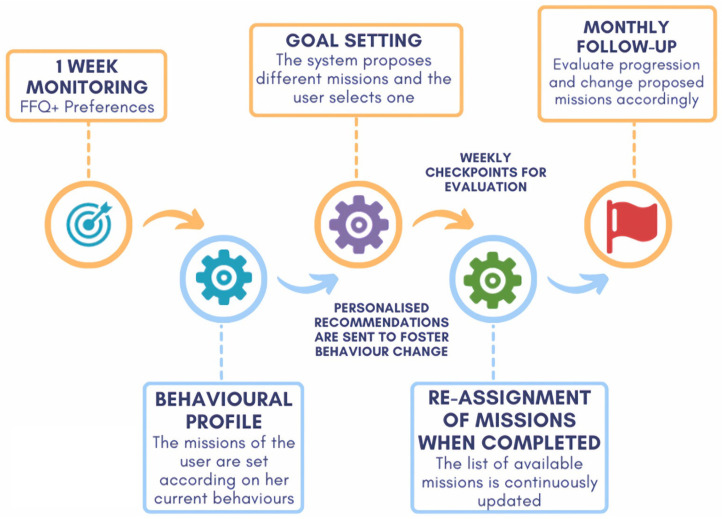
Behaviour Change Workflow.

In more detail, the system’s process, which is based on the HAPA paradigm, is shown in Figure 1. Over the course of a week, we gather user information through several standard questionnaires that are specially adapted by nutritionists and designers for use in a mobile phone interface. We create a behavioural profile using these data, which allows us to tailor personalized dietary missions to each individual. The user is then invited to choose one of the suggested missions to focus on for the following week(s). Once a mission is selected, the intervention designed to modify the behaviour connected to the chosen mission starts. The intervention involves giving them various kinds of individualized recommendations to assist in reaching their objectives and changing their behaviour. The user can change missions at any moment by simply selecting a different one if they becomes stuck, bored, or just wants to check another type. During the process, the user’s behaviour can be monitored at several checkpoints to evaluate the progression and to reprogram missions if necessary.

### 2.2. Dietary Assessment and User Profiling

To assess the dietary habits of the user, a custom-made food frequency questionnaire (FFQ) was developed. The modified-FFQ was designed on the basis of the “Small changes to eat better” food guide and includes 20 different foods or food groups and a list of representative foods and portion sizes (by units or household measures) within each food group. Through the modified FFQ, the user is asked to report the average eating frequency (monthly, weekly, or daily) of each food group over the previous 3 months. The modified FFQ is available in Table S1. In order to tailor the recommendations to specific diets, a questionnaire about dietary habits is also used. Through this questionnaire, the users can indicate whether or not they are coeliac and/or if they follow a vegetarian diet and what type (vegan, ovo-vegetarian, or lacto-ovo-vegetarian).

With these two blocks of information being taken into account, the nutritional behaviour profile is built. The goal of profiling is to develop a conceptual understanding of the user. The primary objective of this method is to modify and adapt the system to meet the unique needs of the users. In order to do this, the system collects the particular data that result from the processing of the aforementioned questionnaires, which are then aggregated to produce a behavioural status perspective of the individual. This is done to improve the coaching supplied to the end user as well as the system reaction.

### 2.3. Design of a Gamified Environment

In order to foster adherence and promote behaviour change, the solution includes gamified elements that are presented to the user through missions to be accomplished, challenging the user to achieve key dietary goals in an engaging and stimulating way. This strategy also considers goal setting as a key BCT that facilitates the acquisition of new habits since the user is asked to select and work in only one mission at a time while each mission is divided into three levels in order to segment small dietary changes into steps and to integrate them into their daily lives.

The dietary missions were designed also taking into account the guidelines “Small changes to eat better” and were developed in close relation to the CarpeDiem FFQ. In total, 39 missions were designed relating to 13 different food groups (3 levels for each food group). For each mission, a title and a brief description is provided, including an explanation of the mission, nutritional benefits, and encouraging words. In Table 1, an example of the missions linked to favour the intake of nuts is given.

To encourage user engagement and adherence, a three-layer coaching timeline is suggested. This multilayered system enables users to (1) select a goal (mission) from the three most relevant that the system has identified through an analysis of the modified FFQ responses, (2) be categorized into one of the three mission levels (beginner, advanced, or expert), and (3) complete the mission by carrying out the activities that the system has recommended in the form of recipes, activities, or dietary recommendations. This makes CarpeDiem an approachable framework that transforms broad objectives into precise actions supporting their accomplishment and, consequently, the fulfilment of users.

#### Mission Monitoring

Nutritional tracking is focused only on the food group that the user is aiming to improve through the mission at the time and does not attempt to compute the nutrients of every meal. To achieve this, the system’s gamification features are leveraged, and user compliance with the chosen mission is monitored to measure food group consumption. For instance, during the weeks when the mission “Innovate your sandwiches and toasts to reduce the consumption of cold meat” is active, only the number of healthy options the user has consumed in place of sandwiches with cold meat is tracked. To accomplish this, the system prompts the user to press a button each time a healthy sandwich is consumed, while aiming to be as undemanding as possible. A shorter version of the modified FFQ that captures a 1-month progression is then transmitted once more to update missions and levels in accordance with the user’s new behaviours in order to check the progression of the food categories on a higher level. This gives the system an additional layer to verify that the behaviour change actually occurred and is sustainable in time.

### 2.4. The Nutritional Recommender System

The Nutritional Recommender System (NRS) based on the BECOME platform [16] can be thought of as CarpeDiem’s intelligence. Its objective is to develop the data analysis components necessary to provide users individualized recommendations and feedback based on combined data from the questionnaires. For action planning, the NRS helps users in changing their eating behaviour, scheduling meals, creating their grocery list, and making healthier food choices, with the aim of promoting a healthier lifestyle.

The NRS controls the required actions that must be carried out by the user differently based on a given situation in an effort to avoid being overbearing and repetitious. In this sense, it carefully decides what recommendations should be made during the intervention to modify the behaviour chosen by the user through the mission.

#### 2.4.1. Scoring System and Mission Selection

Through the gamified nature of the system, the user is categorized into one specific level inside the mission. To automate this decision, a scoring system is implemented as part of the NRS to automatically indicate the user’s level for each of the food groups. To do so, the results of the profiling phase are analysed using a rule-based reasoning (RBR) approach to determine (a) the level (beginner, advanced, or expert) the user is in for each of the target food groups and (b) a ranked list of missions to be proposed to the user based on the calculated levels and the food preferences marked on the questionnaires.

RBR systems are one of the oldest systems that allow reasoning over predefined knowledge. They are useful for representing the knowledge of domain experts in a software system. All of this knowledge is represented by IF–THEN–ELSE rules. However, determining rules is typically difficult. For this, we use a different field called knowledge representation, another branch of AI that deals with knowledge modelling and manipulation.

In CarpeDiem’s scoring system, the RBR approach is used with the twofold purpose of representing expert’s knowledge (i.e., the food groups, their associated missions, and the scores), and chooses from a list of the available “Mission+Level” options that are provided to the user as ranked alternatives to choose from.

The RBR engine has the following components:

- a working memory (WM), which contains the objects representing the user profile derived from the analysis of the questionnaires;
- a rule base (RB) conformed to by all the rules needed to represent the expert’s knowledge and the actions to be triggered; and
- an inference engine (IE), which is a component used to deduce new knowledge from existing knowledge and rules.

Facts are stored in the WM, which contains the objects needed for taking decisions. It incorporates current information about users that can be added, removed, and modified. The user profile data are collected and updated in the WM through the IE when rules are triggered. The IE continuously analyses if there is any potential event to be evaluated (e.g., a mission is completed, and thus new missions should be proposed). After this, a proper rule from the RB is selected and fired through the IE to generate a response.

Based on the food groups of interest, we searched for normal values and the cutoffs relative to each of them. The punctuation used as the bases of the scoring system was established by means of the recommended frequency of servings for each food group provided in the guidelines “Small changes to eat better”. A higher score was given when the frequency of consumption was distant from the recommendations, and a lower score was given when it was close to the recommendations. A maximum score of 10 was assigned to some food groups that were considered more relevant according to their beneficial or detrimental health effects (vegetables, water, sugary foods, red and processed meat, and ultraprocessed foods), when the frequency of servings was very distant from the recommendations (i.e., more than seven servings/week of processed meat). Following this line, fruits obtained a maximum score of 9 and all the other groups a maximum score of 8.

All this information is represented in the system so that when the NRS detects that a new scoring should be performed because there is new information on the user profile, the scoring system is triggered to calculate the score relative to each food group (e.g., fruits: 10 points; vegetables: 6 points; nuts: 3 points), and then food groups are ranked from a higher to lower score, meaning that the food groups categorised with a 10 will be in a higher position in the ranking. For simplification and with the aim of transforming it to the gamified levels, food groups come with a three-level scale to measure the users’ status per food group as follows: 1, poor; 2, average; and 3, good. Thus, the scores are grouped to cover these three levels (e.g., in level 1 of food group *Nuts*, all the scores higher than 5 would be entered).

This process results in a ranked list of “Food Group + Level”, which is then translated into the corresponding missions (see Table 1 as a reference with the example of the missions related to *Nuts* Food Group). The first three missions of the resulting list are then chosen by the system and displayed to the user in the interface (see Section 3). The user can therefore decide which of these to begin working on the next week(s). Every mission has a unique time limit (such as one week) in which it is suggested to be accomplished, and the user is guided through the aforementioned recommendations to gradually complete the objective. The user is informed of the level improvement if he or she completes the mission; otherwise, if the user is stuck, he or she is informed and has the option to switch missions.

#### 2.4.2. The Nutritional Motivational Repository

At CarpeDiem’s Nutritional Motivational Strategies Repository, there are numerous methods that have been shown to improve quality of life (QoL), notably health-related quality of life (HRQoL), in chronic diseases. These strategies establish a set of more than 500 recommendations that are prepared to allow users to be customized and personally tailored depending on their preferences, as well as to monitor a number of indicators with proven significance. The repository includes concepts, recipes, activities, and suggested actions to promote behaviour change in nutrition, and it is based on the knowledge of nutritionists who are subject-matter experts in the field, on international standards (such as those set by the World Health Organisation), and on evidence-based literature to establish referenced cutoffs. The following types can be used to categorize the strategies:

- Did you know…? tips: Some statements providing motivations for healthy eating were developed, including information about nutritional and health benefits or detriments associated with the consumption of food within each food group. This type of recommendation that enhances education in behavioural change is crucial for promoting long-term behaviour change. By providing individuals with clear and actionable information about the benefits and drawbacks of specific behaviours, they are better equipped to make informed decisions and adopt healthier habits. Furthermore, this type of education can help to dispel myths or misconceptions about healthy behaviours, which might have previously discouraged individuals from making positive changes.
- General recommendations for healthy eating: The recommender system also offers general advice on healthy dietary habits not associated with the missions. By providing tips and guidance on healthy cooking techniques, meal planning, and mindful eating, the system helps users to build skills and increase their knowledge about healthy behaviours. This can enhance motivation by providing users with a deeper understanding of the benefits of healthy eating to subsequently make healthy choices a regular part of their daily routine.
- Recommendations linked to missions: To help and motivate the user achieve the missions, a set of dietary and culinary recommendations was developed for each mission. Recommendations included tips, practical ideas, suggestions, and recipes (some of them linked to specific websites). In this context, a total of 390 recommendations were generated. All the recommendations were tagged according to their suitability to the different dietary preferences (vegan, gluten-free…), while some specific recommendations for each dietary preference were also included. Each of the recommendations was provided with an identifier, a general title, a sentence, and the associated food group and mission. Moreover, when relevant, some recommendations were assigned to a specific time.

In this context, the nutritional recommendations appear as follows. After users select a mission, the NRS provides them with a *Did you know…?* tip related to the food group of the mission. Subsequently, the users are given one recommendation related to the chosen mission every two days and one general recommendation about healthy behaviour every week to help them stay motivated.

## 3. Results

### 3.1. Design and Development of the User-Centred Mobile Application

The nutritional framework for behavioural change using the HAPA Model provides the conceptual foundation for promoting healthy dietary behaviour. However, it is not enough to simply define this theoretical approach; its practical application through the development of a user-centred mobile application is equally important. The mobile app developed in CarpeDiem is designed to incorporate the key components of the nutritional framework and the HAPA model, with a focus on reducing user interaction and enhancing user experience. The success of this app is will not only be determined by its approach for promoting sustained healthy behaviour change but also to the crucial role of user-centred design in fully unlocking the framework’s potential and ensuring its accessibility and usefulness to a diverse range of users. In this section, we will present the results of the development process for this application, highlighting its features and functionalities and demonstrating its potential impact on promoting healthy dietary behaviours.

**Figure 2:**
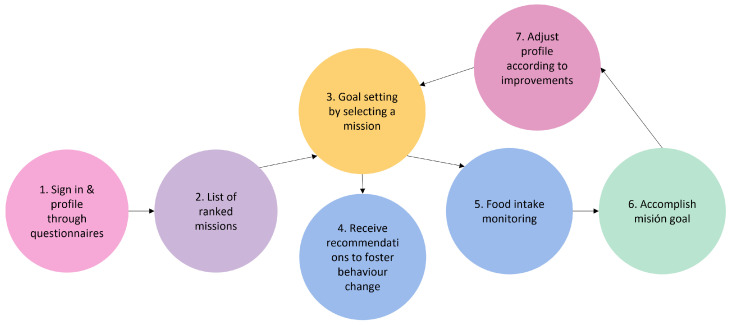
Workflow of CarpeDiem app’s nutrition pillar.

**Figure 3:**
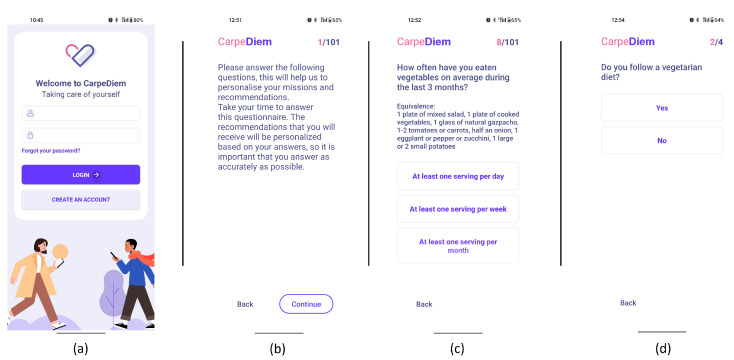
Screenshots from the CarpeDiem app showing user sign-in and questionnaire profiling. (**a**) User login. (**b**) Questionnaire information. (**c**) Modified FFQ questionnaire. (**d**) Dietary preference questionnaire.

**Figure 4:**
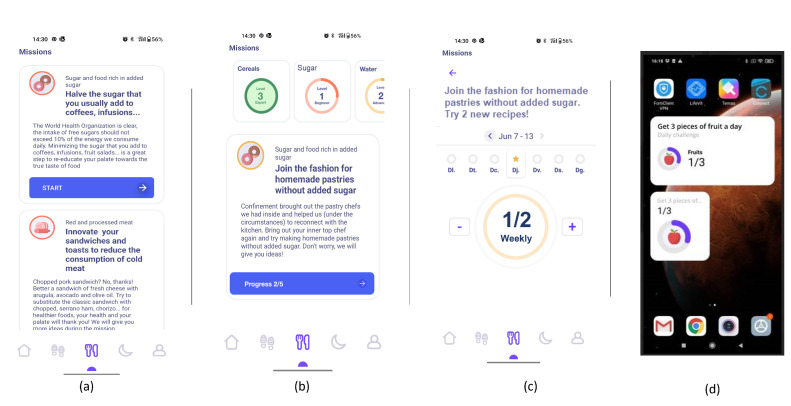
Screenshots from the CarpeDiem app showing missions interface. (**a**) List of missions proposed. (**b**) Missions overview. (**c**) Mission logging. (**d**) Mission logging through a widget.

**Figure 5:**
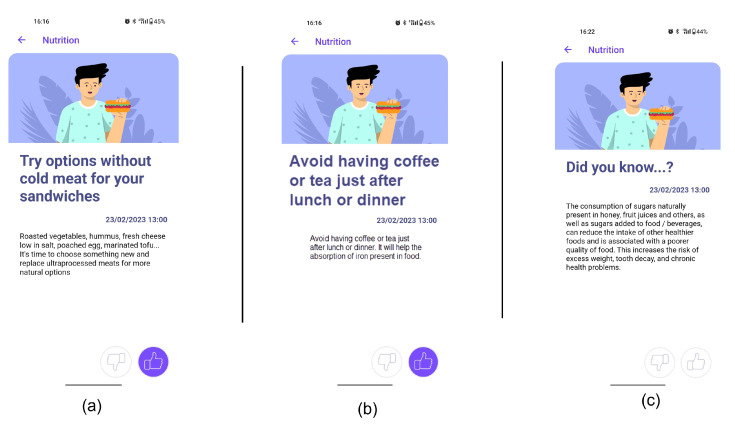
Screenshots from the CarpeDiem app showing different types of recommendations. (**a**) Recommendation linked to mission. (**b**) General recommendation for healthy eating. (**c**) Did you know…? tip.

The front end of CarpeDiem is a smartphone app with a cutting-edge user interface that helps and inspires users while educating them on healthy behaviours, setting alarms and nudges, and encouraging them to adhere to individualized goals. It has individualized interfaces including the ability to visualise daily progress, display scores, show personalised recommendations, or ask users whether they like or dislike proposed activities. The primary elements of CarpeDiem’s nutrition pillar are listed in Figure 2, along with the sequence of the users’ interactions with the app. The following is the primary workflow:

1. Sign-in and profiling through questionnaires. After th uses signs in using the interface depicted in Figure 3-screenshot (a) and screen (b) appear to explain the value of responding to the following questionnaires. The user is then prompted to complete the modified FFQ (screenshot (c)) and dietary preference (screenshot (d)) questionnaires.
2. List of ranked missions. The responses of the questionnaires are used by the NRS to propose to the user a rank of missions and the level that the user should work on. In Figure 4-screenshot (a) and (b), we show three missions that have been proposed to a user.
3. Goal setting by selecting a mission. When the user decides which mission he or she wants to start working on, the user presses the blue button shown in Figure 4-screenshot (a), and the intervention begins. If the user becomes blocked, bored, or simply wishes to change missions, this can be done at any time by just choosing another one.
4. Receipt of recommendations to foster behaviour change. Once there is a selected mission, the NRS starts sending recommendations to change the behaviour of the user. The most suitable recommendation for the user is selected from the Nutritional Motivational Repository. In Figure 5, we show the interface of three different types of recommendations. The recommendations, delivered to the user in the form of push notifications are helpful in reminding the user without being overly invasive. The number of recommendations received throughout the week was 3–4.
5. Food group intake monitoring. In Figure 4-screenshot (c), we show the interface used to log the food intake linked to the mission. In the example, the user just needs to press the *+* button when trying one new home-made pastry recipe proposed in the recommendations of the mission. By using widgets added to the main screen of their mobile phone (see Figure 4-screenshot (d)), the user can conveniently track their missions, minimising the number of actions they need to take and thus facilitating the monitoring of the mission.
6. Accomplish mission goal. When a mission is completed, the user is given the option to level up. It means that a higher-level mission from the same food group is proposed. If the use has already completed the highest level mission, the NRS suggests another mission from a different food group based on the results of the scoring system.
7. Profile adjustment according to improvements. Once missions are completed, the profile is modified in accordance with the improvements, resulting in tailored recommendations.

### 3.2. HAPA Phases and Strategy Implementation

The proposed design is aligned with the different phases of the HAPA model (motivation and volition). To achieve this alignment, we have incorporated specific characteristics into our system that correspond to the strategies and interventions recommended for each phase. In this subsection, an explanation on how these strategies were implemented is provided. By doing so, we aim to illustrate how the framework effectively supports users in their efforts to initiate and maintain healthy behaviours.

#### 3.2.1. Motivational Phase

- Risk perception: The system provides informative nutrition recommendations of the “did you know…?” type. Such recommendations enhance education by providing individuals with clear and actionable information about the benefits, drawbacks, and risks of specific behaviours, so they are better equipped to make informed decisions, increase awareness, and adopt healthier habits.
- Outcome expectancies: The system provides general recommendations not associated with the missions. This can enhance motivation by providing users with a deeper understanding of the benefits of healthy eating (healthy cooking techniques, meal planning, and mindful eating) to subsequently make healthy choices a regular part of their daily routine.
- Self-efficacy: When users starts a mission, the system sends several recommendations in a specific order to help them build the skills and knowledge required to achieve their behaviour change goal. This approach ensures that users have the necessary support and guidance to successfully make the desired change, which in turn can increase their confidence in their ability to modify their behaviour for the better.
- Intention formation: During a defined timeframe of one to two weeks, our system encourages users to focus solely on one food group at a time. By centring their efforts on modifying one specific aspect of their diet, users are less likely to feel overwhelmed. This approach allows individuals to make gradual and sustainable changes to their eating habits, without feeling like they have to overhaul their entire diet all at once.

#### 3.2.2. Volitional Phase

- Planning: The system categorizes the user into one of three mission levels (beginner, advanced, or expert), each with a set of realistically achievable goals that progressively increase in complexity towards the final objective. This approach allows users to start with simpler goals that are appropriate for their skill level and gradually work toward more challenging ones.
- Coping planning: The system provides users with feedback in the form of notifications if they are not making progress toward completing their mission. These reminders are designed to help users stay on track and motivated to achieve their behaviour change goals. Additionally, the system recommends alternative missions and provides them with options to choose from. This approach allows users to select a mission that is better suited to their preferences and abilities, which can increase their chances of successfully achieving their desired behaviour change.
- Action control: The system monitors both adherence to the mission and objective adherence to changes in all food groups using a monthly adapted FFQ. By assessing changes in other food groups that may be indirectly affected by improvements in healthy habits, a more accurate track of the overall impact of behaviour change on an individual’s diet is achieved. This approach allows for the acquisition of a comprehensive understanding of how changes in one area of diet and behaviour can have a ripple effect on other areas, which can inform future recommendations and interventions.
- Feedback: The mission screen displays the user’s current level for each food group. Whenever the user completes a mission, the level for that particular food group changes accordingly. To acknowledge user progress, the user receives a congratulatory message upon levelling up.

## 4. Discussion

In this paper, we present the design and development of the mHealth framework for nutritional behaviour change, the main component of the CarpeDiem mHealth app, aimed at the promotion of healthy habits among the general population by working on the three main pillars of health: nutrition, physical activity, and sleep. The nutritional framework is based on a gamified system that delivers personalized dietary missions to the user, which are focused on key food groups, and provides motivational recommendations that help the user to achieve these missions. As far as we know, this is the first app presenting these characteristics.

The crucial role that diet plays in our long-time health is well-known [35]. Nutritional status is strongly related to health, and suboptimal diets have become one of the most preventable risk factors for noncommunicable diseases (NCDs) such as obesity, diabetes, or hypertension [36,37]. Recent data showed that 11 million deaths worldwide were attributable to dietary risk factors in 2017 alone [35]. Furthermore, the number of overweight or obese individuals has increased alarmingly over the years, and forecasts estimate an increase greater than 20% in obesity prevalence worldwide by 2030 [38,39]. Obesity is in turn closely associated with a higher risk of developing other metabolic disorders, such as hypertension. The prevalence of hypertension has doubled globally from 1990 to 2019, with an estimated 1.28 billion adults aged 30–79 years affected by this condition [37]. In addition, hypertension and diabetes are the main causes of chronic kidney disease (CKD), whose global prevalence in 2017 was 9.1% [40]. These staggering figures suggest an urgent need to enhance dietary habits and lifestyle among the population, as the impact of these NCDs is not only on individual health but also on healthcare systems and the economy.

The CarpeDiem app differs from the existing nutritional apps by the use of goal-setting as a main BCT that allows the user to achieve small and realistic dietary changes progressively and thus to gradually incorporate them into daily life, generating consolidated habits. Typically, nutritional apps rely on the change of many aspects of the diet at a time, pushing the users to readapt most of their dietary habits in a short period of time. This has been associated with a lack of adherence in the long-term since it is hard to maintain all these changes over the time when they have not been acquired as a habit. Moreover, in CarpeDiem, users can select the preferred mission to start and are also able to change it at any time, giving them a sense of control that has been shown to be critical for behaviour change. Lastly, the integration of the HAPA model in the scientific design of the present technological solution represents a strength of this mHealth app, offering an evidence-based approach for changing dietary behaviour in an effective and sustained way.

Personalization is another key characteristic of the present nutritional framework. The one-size-fits-all approach has been demonstrated to have many limitations and to be ineffective in terms of inducing adherence and motivation, thus reducing the possibility of achieving health goals. In this sense, personalised nutrition tries to overcome the interindividual differences by developing targeted and customised interventions adapted to the specific and unique characteristics of each person. In CarpeDiem, we provide ad hoc missions and recommendations based on both users’ food intake and dietary preferences. Finally, it is important to highlight the fact that CarpeDiem will be used not only as an intervention tool but also as a data collection instrument through the answers to the questionnaires, the recording of the progress of the missions, the logs on the use of relaxing sounds, and the data obtained through the activity tracker. The data collected from thousands of app-users can be analysed using data mining techniques to detect patterns, associations, and relationships between datasets, which can then be used to develop predictive models [41]. Public health authorities can leverage this information to gain insights into public health trends, as well as to inform policy decisions and health interventions. Moreover, the characterisation of the population can provide directions to researchers about what kind of intervention/health problem should be primarily addressed [42].

Although the features of the mHealth app developed in the current work are based on scientific evidence and have been developed by healthcare professionals, its feasibility and usability needs to be evaluated in real users; it must also be scientifically validated in order to determine the efficacy of this app for improving eating habits in the short- and long-term and to determine this can translate into better health outcomes. Thus, a large randomised controlled trial, including a period of follow-up, must be conducted to confirm our hypothesis. The primary objective of the study would be to assess the usefulness of the app to achieve a higher adherence to the Mediterranean lifestyle pattern evaluated by the Mediterranean Lifestyle (MEDLIFE) index. For this, a parallel, randomised controlled study design would be used. Adult participants would be randomised in two groups: control and intervention. Participants from the intervention group would use the CarpeDiem app and the Fitbit brand activity bracelet. In contrast, participants from the control group would use the Fitbit brand activity bracelet and receive general recommendations for healthy lifestyle habits. Participants would be asked to use the app or follow the general healthy lifestyle recommendations for 6 months. Secondary outcomes would be blood pressure, anthropometric variables (BMI, waist circumference, body composition), food intake and dietary diversity (analysed through the Predimed food frequency questionnaire), physical activity (obtained via the IPAQ questionnaire), sleep quality (obtained via the PSQI questionnaire), quality of life (obtained via the QOLQ questionnaire), and user experience (obtained via the UEQ questionnaire). Three visits would be conducted (at the initial point, after 3 months, and after 6 months). If we were able to confirm our hypothesis, the same framework could be adapted for specific populations, such as older people or pregnant women, and also to particular conditions, such as prevention of cardiovascular disease.

The CarpeDiem app could be prescribed by healthcare professionals but also introduced in schools and workplaces as a way to promote preventive behaviours. Ultimately, it could have a significantly positive impact on the public health system in several ways. Since digitalisation has assumed a massive role in ascertaining the status of modern society, this kind of approach, i.e., mHealth platforms, provides a significant benefit to health education and health policy, as it allows for the easier dissemination of information to the entire population. Technology has the potential to be an effective tool in improving public health outcomes. In fact, the European Public Health Association (EUPHA) has enabled a new Digital Health section to canalize technological efforts toward an improvement of public health in Europe [43]. By adopting self-management practices, such as monitoring one’s health status, adhering to treatment plans, and engaging in healthy behaviours such as regular exercise and a balanced diet, individuals can assume an active role in their healthcare [44,45]. This can lead to improved health outcomes and general well-being, potentially reducing the burden of disease and lowering healthcare costs [46]. Additionally, by promoting healthy behaviours and self-management, healthcare systems can focus their resources on other areas of need, enhancing their capacity to provide high-quality care. Moreover, there is substantial evidence that individuals who practice healthy habits tend to have higher levels of energy, greater focus, and improved cognitive function than those who do not engage in such behaviours [47,48]. This can be translated into increased productivity and output, which are critical drivers of economic growth.

In our paper, several limitations must be addressed. Firstly, as explained, the lack of piloting is a significant limitation. Comprehensive testing should involve various user groups, such as the general population, elderly users, individuals with health problems such as obesity, and mental health patients. Another limitation is that all the required data are self-reported and therefore subject to recall and social desirability bias. Our system’s reliance on forms for data collection may result in incomplete or inaccurate information provided by users and also lead to a lack of motivation in the long-term. To address this, we could explore alternative and smart data collection methods or develop more engaging and user-friendly systems. Moreover, our current recommender system does not tailor to specific dietary needs, such as food-allergic or diabetic individuals. Future research should explore personalising the experience for these user types to make the system more inclusive and adaptable.

In conclusion, we have designed and developed an mHealth framework for nutritional behaviour change that has been integrated into the CarpeDiem mHealth app. This framework is mainly characterised by the implementation of the HAPA model, a psychosocial theory of health behaviour change, which guided its design, the use of a gamified system based on personalized dietary missions that have been developed to incorporate the dietary guidelines published by the Public Health Agency of Catalonia, a dietary assessment and setting of objectives based on food groups intake instead of calories and nutrients, the personalization of the system, and the use of a recommender system based on advanced AI techniques. This work was completed by an interdisciplinary team, including nutritionists. Considering the above, we think that this approach could foster a sustained improvement of eating habits over time among the general population, which is the main challenge of dietary interventions, and thus could help to decrease the risk of individuals developing chronic conditions associated with unhealthy dietary habits and facilitate a better quality of life. Nevertheless, the efficacy of the present mHealth nutritional framework must be validated in well-designed, future scientific studies. If its efficacy is proven, the presented intelligence tool could be prescribed by healthcare professionals and be introduced in schools and workplaces as a way of bringing the dietary guidelines closer to the community.

## Figures and Tables

**Table 1:** Three levels of missions related to the food group *Nuts*.

Mission Name	Description	Type of Users
Nuts Level 1: Fill your pantry with nuts	They are an ideal snack and still go unnoticed by most people. Nuts, such as walnuts, almonds, hazelnuts, pistachios, and cashews, will surprise you with their beneficial properties. Fibre, healthy fats, proteins, vitamins, minerals, and other protective substances make up this great food	Users who consume 0/1/2 servings of nuts per week
Nuts Level 2: Incorporate nuts into your diet at least 3 days a week	Did you know that despite the fact that nuts are rich in calories, their consumption has not been associated with weight gain? Therefore, you should not worry and incorporate them as part of a daily balanced diet. A daily handful is a very good place to start. You can add them in the bowl of yogurt, put them in salads, and eat them as an afternoon snack or in the middle of the morning; the possibilities are endless!	Users who consume 0/1/2 servings of nuts per week and have already completed Nuts Level 1
Nuts Level 3: Be creative and try new recipes that incorporate nuts	It’s time to go a little further and test your culinary abilities. Try experimenting with new culinary preparations if you’re bored of having a handful of nuts with yogurt or as a mid-morning snack. Your creativity is the only limit!	Users who consume more than 3 servings of nuts per week

## Data Availability

Not applicable.

## Supplementary Materials

The following supporting information can be downloaded at: https://www.mdpi.com/article/10.3390/nu1010000/s1, Supplementary Table S1: CarpeDiem modified Food Frequency Questionnaire (FFQ).
