# Supplementary material for: A Tailored and Engaging mHealth Gamified Framework for Nutritional Behaviour Change
Source: Nutrients. 2023 Apr 18;15(8):1950. doi: 10.3390/nu15081950 (PMC10142076; doi:10.3390/nu15081950)
Supplement: Supplementary file 1 [file nutrients-15-01950-s001.zip › nutrients-2283660-supplementary.pdf]

Table S1. CarpeDiem modified Food Frequency Questionnaire (FFQ).

Food Groups	Foods items	Portion sizes	Never or almost never	Per month			Per week						Per day					
				1	2	3	1	2	3	4	5	6	1	2	3	4	5	≥6
Fruits	Apple, pear, orange, tangerine, strawberries, cherries, grapes, peach, banana, melon ...	1 medium piece, 2 tangerines, 1 cup of cherries, strawberries ..., 1 slice, 1 cluster or dessert plate																
Vegetables	Onion, tomato, lettuce, zucchini, aubergine, leek, green bean, carrot, pepper, artichoke, spinach, cauliflower, mushrooms .. potatoes ..	1 plate of mixed salad, 1 plate of cooked vegetables, 1 glass of natural gazpacho, 1-2 tomatoes or carrots, half an onion, 1 eggplant or pepper or zucchini, 1 large or 2 small potatoes																
Legumes	Lentils, chickpeas, beans, peas ...	1 individual normal plate																
Nuts	Walnuts, almonds, hazelnuts, pistachios, pine nuts, cashews, peanuts ... (raw or lightly toasted)	1 handful or individual serving																
Whole grains	Pasta, rice, couscous, bread ... wholemeal<br>Oatmeal or corn flakes<br>DOES NOT include sugary whole grain cereals or cookies (Fitness, Special K ...)	1 regular plate, 3-4 slices or a bread roll, 30 grams of oatmeal																
Sugary and flavoured yogurts	Sugary/sweetened yogurts, flavoured yogurts, ...	1 unit																
Milk with added sugar	Milk/latte with added sugar or sweetened cocoa .. milk shakes ...	1 cup																
Dairy desserts	Custard, flan, rice pudding, ice cream ...	1 individual portion																

**Fish and seafood**	Hake, cod, grouper, sole, whiting ...<br>Sardines, tuna, bonito, mackerel, salmon, anchovies ...<br>Squid, cuttlefish, octopus, baby squid ...<br>Prawns, shrimps .. Clams, mussels .. (includes fish and shellfish canned in water and/or in oil)	1 individual fillet, 4-5 pieces (prawns ..), 6 units (clams, mussels ..), 1 small or half normal can																
**Red meat**	Beef or veal, pork, lamb, horse meat.	1 small steak																
**Processed meat**	Burgers, meatballs, sausages, blood sausage, bacon ...	1 hamburger, 3 meatballs ..																
**Cold cuts**	Salami, chorizo, mortadella, ham, serrano ham ...	2-3 slices of cold cuts ..., a slice of ham																
**Virgin olive oil**	Extra virgin or virgin olive oil	One tablespoon																
**Sugar and food rich in added sugars**	Honey, white or brown sugar, panela .. Jams ..<br>Pastries: Maria type cookies with or without chocolate, fiber or whole grain cookies, donuts, churros, chocolates and bonbons (≤70% cocoa), nougats, cereal bars, cakes, sponge cakes, muffins, croissants or other industrial pastries	1 teaspoon of honey or other sugar, 4-6 units (cookies), 50 gr pastries and cakes, 1 unit croissant or other industrial pastries																
**Beverages rich in added sugars**	Packaged fruit juices, cola drinks, lemonades, tonics, other carbonated drinks with sugar, energy and isotonic drinks...	1 glass or bottle																
**Salt**	Salt	1 pinch																
**Salty foods**	Bread, crispbreads, cold cuts, cheese, potato chips and similar, salty nuts, olives, preserves, ready meals...	2 small slices (50g), 4 crispbreads, 1 slice of ham or 2 slices of cold cuts, 2 slices of cheese, 1 individual bag of chips, 1 handful or commercial portion of salty nuts, 10 olives, 1 small can, 1 commercial serving (ready meals)																

**Ultraprocessed foods**	Foods with a very long list of industrial ingredients (for example, more than 3 ingredients), with little or no amount of fresh/staple foods:<br><br>Pre-cooked pizzas, pre-cooked dishes, sauces and meat or vegetable extracts, instant soups, ..	1 portion of pizza, 1 croquette or other similar precooked, 1 plate of creams and soups, 1 teaspoon of sauce																
**Water**	Water, sugar-free infusions, broth	1 glass or bottle																
**Alcoholic beverages**	Wine, cava, beer, spirits, anise, whiskey, vodka, gin, cognac	1 glass, 1 jug, 1 cup																
